# Bound Polyphenols from Red Quinoa Prevailed over Free Polyphenols in Reducing Postprandial Blood Glucose Rises by Inhibiting α-Glucosidase Activity and Starch Digestion

**DOI:** 10.3390/nu14040728

**Published:** 2022-02-09

**Authors:** Yu Zhang, Bing Bai, Yu Yan, Juan Liang, Xiao Guan

**Affiliations:** 1 School of Health Science and Engineering, University of Shanghai for Science and Technology, Shanghai 200093, China; zyu20@usst.edu.cn (Y.Z.); Baibing0714@126.com (B.B.); yanyu981622@163.com (Y.Y.); nxlj5200@163.com (J.L.); 2 National Grain Industry (Urban Grain and Oil Security) Technology Innovation Center, Shanghai 200093, China

**Keywords:** bound polyphenols, quinoa, α-glucosidase, T2DM, postprandial glucose

## Abstract

Inhibiting α-glucosidase activity is important in controlling postprandial hyperglycemia and, thus, helping to manage type-2 diabetes mellitus (T2DM). In the present study, free polyphenols (FPE) and bound polyphenols (BPE) were extracted from red quinoa and their inhibitory effects on α-glucosidase and postprandial glucose, as well as related mechanisms, were investigated. HPLC-MS analysis showed that the components of FPE and BPE were different. FPE was mainly composed of hydroxybenzoic acid and its derivatives, while BPE was mainly composed of ferulic acid and its derivatives. BPE exhibited stronger DPPH and ABTS antioxidant activities, and had a lower IC50 (10.295 mg/mL) value in inhibiting α-glucosidase activity. The inhibition kinetic mode analysis revealed that FPE and BPE inhibited α-glucosidase in a non-competitive mode and an uncompetitive mode, respectively. Furthermore, compared to FPE, BPE delayed starch digestion more effectively. BPE at 50 mg/kg reduced postprandial glucose increases comparably to acarbose at 20 mg/kg in ICR mice. These results could provide perspectives on the potential of BPE from red quinoa, as a functional food, to inhibit α-glucosidase activity, delay postprandial glucose increases and manage T2DM.

## 1. Introduction

According to the World Health Organization, the number of people with diabetes rose from 108 million in 1980 to 422 million in 2014. In 2019, an estimated 1.5 million deaths were directly caused by diabetes [1]. Type-2 diabetes mellitus (T2DM) makes up about 90% of cases of diabetes. Glycemic control is considered as an effective way to manage T2DM, and prolonged carbohydrate digestion is beneficial for T2DM patients. α-glucosidase secreted in the small intestine catalyzes the end phase of disaccharides digestion to monosaccharides. Thus, inhibiting α-glucosidase activity serves as an effective strategy in delaying the digestion of carbohydrate in the gut and reducing the postprandial blood glucose peak [2]. Some clinical drugs aimed at inhibiting the activity of α-glucosidase have been invented, such as acarbose, miglitol and voglibose. However, long-term use of clinical α-glucosidase inhibitors often causes some side effects, such as abdominal discomfort and flatulence [3]. Thus, natural food-derived substances with α-glucosidase inhibitory activity show superiority over some clinical drugs due to their safety and reduced risks of side effects.

Polyphenols, as a major subcategory of phytochemicals, constitute an indispensable part of our daily diet. Polyphenols from various sources, such as tea [4], berries [2] and cereal grains [5], have been reported to show α-glucosidase inhibitory activity and, thus, reduce glucose absorption in the gut. However, most of the studies merely focus on the free polyphenols that are directly extracted from organic solvents. A large part of non-extractable bound polyphenols is ignored, which leads to an underestimation of the total polyphenols content and nutritional value of foods. Bound polyphenols are bound to cell wall components, such as dietary fibers, via chemically covalent bonds, hydrogen bonding and hydrophobic interactions [6]. After solvent extraction, bound polyphenols can be extracted from the remaining solid residues through alkaline/acidic/enzymatic hydrolysis. Cereal grains are important sources of dietary polyphenols, and studies reported that the proportion of bound polyphenols in cereal grains (~60%) was higher than those in fruits and vegetables (~24%) [7].

Quinoa (*Chenopodium quinoa* Willd.), native to the Andean regions of Chile, Peru and Bolivia, is considered as “golden grain” by local people. The high nutritional value of quinoa, including its ideal protein profile, and rich contents of vitamins and polyphenols, brings many health benefits, such as antioxidant, anti-diabetic and anti-obesogenic activities [8]. It was previously shown that pigmented cereal grains contained higher content of polyphenols than non-pigmented ones [9]. Although previous studies have reported the enzyme-inhibitory activities of quinoa polyphenols, there are still a lack of data pertaining to the comparison of free polyphenols and bound polyphenols from red quinoa in the inhibitory effects and mechanism of α-glucosidase, and their effects on starch digestion in vitro and postprandial glucose in vivo. The present study obtained free polyphenols extract (FPE) and bound polyphenols extract (BPE) from red quinoa, and analyzed their composition via HPLC-MS. Their antioxidant activities, effects on α-glucosidase and related mechanism, and effects on starch digestion were investigated in vitro. Furthermore, the postprandial glucose level and Area Under the Curve (AUC) of mice administrated with FPE and BPE were investigated in vivo. Our results aimed to provide a theoretical reference for the development of functional foods for T2DM, and expand the application of quinoa-related components.

## 2. Methods and Materials

### 2.1. Reagents and Materials

Red quinoa grains were purchased from Yihe Agricultural Products Sales Co., Ltd. (Fanshi, Shanxi Province, China). Methanol and acetonitrile for HPLC analysis were of HPLC grade. 2,2-diphenyl-1-picrylhydrazyl (DPPH), 2,2′-azino-bis-(3-ethylbenzo-thiazoline-6-sulphonic acid) diammonium salt (ABTS), α-glucosidase and maltose were purchased from Sigma-Aldrich (St. Louis, MO, USA). All other chemicals were of analytical grade.

### 2.2. Extraction of FPE and BPE

The extraction of free polyphenols and bound polyphenols from red quinoa grains was conducted according to previous studies with slight modification [10]. Briefly, red quinoa grains (40 g) were ground into powder and extracted with 70% ethanol (1 L). The extraction was carried out on a rotary shaker at 400 rpm for 3 h at room temperature. The mixture was centrifuged at 6000 rpm for 10 min (Thermo Fisher Scientific, Waltham, MA, USA) and the supernatant solution was collected and rotary-evaporated under vacuum. Dichloromethane was then added for liquid–liquid extraction to remove the hydrophobic components from the red quinoa grains. The dichloromethane layer was removed and the organic solvent was rotary-evaporated under vacuum. The solution was lyophilized into dryness and labelled as free polyphenols extract (FPE). Then, the residue (20 g) was first treated with 250 mL of 2 M NaOH for a 4-h hydrolysis at room temperature. The mixture was acidified to pH 2 with 6 M HCl, and then centrifuged at 6000 rpm for 10 min. The supernatant was collected and the extraction process of bound polyphenols extract (BPE) was similar to that of FPE as described above.

### 2.3. Detection of Total Polyphenols Content and Total Flavonoids Content

The total polyphenols content (TPC) was determined using the Folin–Ciocalteu assay. Briefly, 400 μL of sample solution (FPE or BPE dissolved in distilled water) was added to 1 mL of 1 M Folin–Ciocalteu reagent. The mixture solution was diluted to 2 mL and was shaken thoroughly. The mixture solution was kept in darkness for 5 min at room temperature. A quantity of 5 mL of 5% Na_2_CO_3_ (*w*/*v*) was added to the mixture solution and the reaction was maintained for 60 min at room temperature. Later, the absorbance was read at 765 nm. Results were expressed at milligrams per gram of gallic acid equivalents (GAE).

The total flavonoids content (TFC) was determined based on a previous method with slight modification [11]. In brief, 100 μL of sample solution (FPE or BPE dissolved in methanol) was mixed completely with 300 μL of 5% NaNO_2_ and stood for 5 min. Then, 300 μL of 10% Al(NO_3_)_3_ was added to the mixture and stood for 6 min. A quantity of 4 mL of 1 M NaOH was added afterwards and 400 μL of 30% ethanol was added to make a final volume of 10 mL. The mixture was completely vortexed and stood for 10 min. The absorbance was measured at 510 nm. The TFC was expressed as milligrams per gram of rutin equivalent (RE).

### 2.4. Liquid Chromatography Analysis of FPE and BPE

UPLC-UV-ESIMS analysis was performed on a Waters Acquity I-Class platform coupled with a Bruker Esquire 3000 Plus ion trap mass spectrometer (Bruker-Franzen Analytik GmbH, Bremen, Germany). Mass Spectra were achieved by electrospray ionization (negative mode). A Zorbax SB-C18 (Agilent, Santa Clara, CA, USA) column (250 × 4.6 mm, 5 μm) was used for separation. The mobile phase consisted of (A) 0.1% formic acid solution and (B) 95% methanol and 5% acetonitrile. The flow rate was 0.8 mL/min and the column temperature was kept at 45 °C. The elution program was as follows: 0–40 min, 0–80% B; 40–42 min, 80–100% B; 42–44 min, 100% B; 44–50 min, 100–0% B. The injection volume was 10 μL and the UV detection wavelengths were set at 280 nm. Commercial standards were used for the quantification of specific polyphenols of FPE and BPE.

### 2.5. DPPH and ABTS Assays

The DPPH assay was performed as previously reported [12]. In brief, 100 μL of sample solution (FPE or BPE dissolved in ethanol) was added into 3.9 mL of 0.1 mM DPPH in ethanol. The mixture was kept in darkness for 20 min at room temperature. Afterwards, the absorbance was measured at 517 nm. Results were expressed as milligrams per gram of Trolox equivalents (TE).

The ABTS assay was performed as previously reported [12]. Quantities of 25 mL of 7 mM ABTS solution and 440 µL of 140 mM potassium persulfate solution were mixed to form the ABTS+ cation solution, which was kept in darkness for 12–16 h before use at room temperature. The ABTS+ cation solution was diluted with ethanol until its absorbance value was 0.7 ± 0.02 at 734 nm. Then, 100 µL of sample solution (FPE or BPE dissolved in ethanol) was added to the diluted ABTS+ cation solution (3.9 mL). The mixture was kept in darkness for10 min at room temperature. The absorbance was read at 734 nm and the results were expressed as milligrams per gram of TE.

### 2.6. α-Glucosidase Inhibition Assay and Inhibition Kinetic Mode

The inhibitory effects of FPE and BPE were assessed based on previous studies with slight modification [13,14]. A quantity of 25 μL of sample solution was mixed with 25 μL of α-glucosidase (1 U/mL), and the reaction was maintained for 10 min at 37 °C. Then, 25 μL 5mM of pNPG in 0.1 M phosphate buffer (pH 6.8) was added to the mixture and reacted for 10 min at 37 °C, followed by the addition of 50 μL of Na_2_CO_3_ (100 mM) to stop the reaction. The absorbance was read at 450 nm using a micro-plate reader (BioTek Instruments, Winooski, VT, USA). All samples were measured in triplicate. The inhibition rate of FPE or BPE was calculated using Equation (1):

(1) $$\%\;\mathrm{inhibition}\;\mathrm{rate}\;=\frac{\left(A_{control}\;-\;A_{control\;blank}\right)\;-\;\left(A_{sample}\;-\;A_{sample\;blank}\right)}{\left(A_{control}\;-\;A_{control\;blank}\right)}$$

where *A_control_* refers to the absorbance of the buffer with the enzyme; *A_control blank_* refers to the absorbance of the buffer without the enzyme; *A_sample_* refers to the absorbance of FPE or BPE with the enzyme; *A_sample balnk_* refers to the absorbance of FPE or BPE without the enzyme. The IC50 value, meaning the concentration of FPE or BPE required for 50% inhibition of α-glucosidase activity, was used to measure the inhibitor potency.

The inhibition mode of FPE or BPE against α-glucosidase was determined according to the plots of a double reciprocal line (1/*v* versus 1/*S*) using the Lineweaver–Burk model. The concentrations of pNPG ranged from 0.5 to 5 mM, and the concentrations of FPE or BPE were 10 mg/mL and 20 mg/mL. The Michaelis constant (*K_m_*) and maximal velocity (*V_max_*) were obtained from the plots:

(2) $$\frac{1}{v}=\frac{1}{V_{max}}+\frac{K_{m}}{V_{max}}\frac{1}{\left[S\right]}$$

where *v* refers to the initial velocity and [*S*] refers to the concentration of substrate (pNPG).

### 2.7. Effects of FPE and BPE on the Digestion of Starch In Vitro

The effects of FPE and BPE on the digestion of rice starch in vitro were tested based on a previous study with slight modification [13]. Rice starch solution (2% in acetate buffer, pH 5.2) with FPE or BPE (10 mg/mL) was prepared. The mixture was heated at 90 °C to gelatinize for 20 min. The simulated process of starch digestion was started by adding 3 mL of mixed enzymes solution (α-amylase: α-glucosidase = 120:80 U/mL). The reaction took place under 37 °C for 240 min with constant stirring (300 rpm). During the reaction, 500 μL of the reaction solution was removed and terminated using ethyl alcohol at 30, 60, 90, 120, 180 and 240 min. The supernatant was collected after centrifugation, and the glucose content was determined using the GOPOD method. 

### 2.8. Effects of FPE and BPE on Postprandial Glucose in Mice

The effects of FPE and BPE on postprandial glucose in ICR mice were investigated according to a previous study [2]. Five-week-old male ICR mice were kept in 12-h day/night cycles and fed a standard chow diet for 1-week acclimatization. Overnight-fasted mice were administrated with maltose (2 g/kg) and treatment solution (PBS, 25 mg/kg FPE, 50 mg/kg FPE, 25 mg/kg BPE, 50 mg/kg BPE, 20 mg/kg acarbose dissolved in PBS) by oral gavage, and then blood glucose was recorded with a glucose meter (Roche Diagnostics, Shanghai, China) at 0, 30, 60, 90 and 120 min. All animal experimental procedures completely adhered to the Guidelines for Care and Use of Laboratory Animals of Shidong Hospital, Yangpu District, Shanghai, China and were approved by the Animal Ethics Committee of Shidong Hospital, Yangpu District, Shanghai, China.

### 2.9. Statistical Analysis

Results are presented as the mean ± standard deviation (SD) for at least three replicates for each sample. Statistical analyses were performed using the SPSS program, version 17.0 (SPSS Inc., Chicago, IL, USA, 2009). Data were analyzed by ANOVA and significant differences were set at *p* < 0.05, *p* < 0.01 and *p* < 0.001.

## 3. Results

### 3.1. TPC, TFC and Antioxidant Activities of FPE and BPE

**Figure 1:**
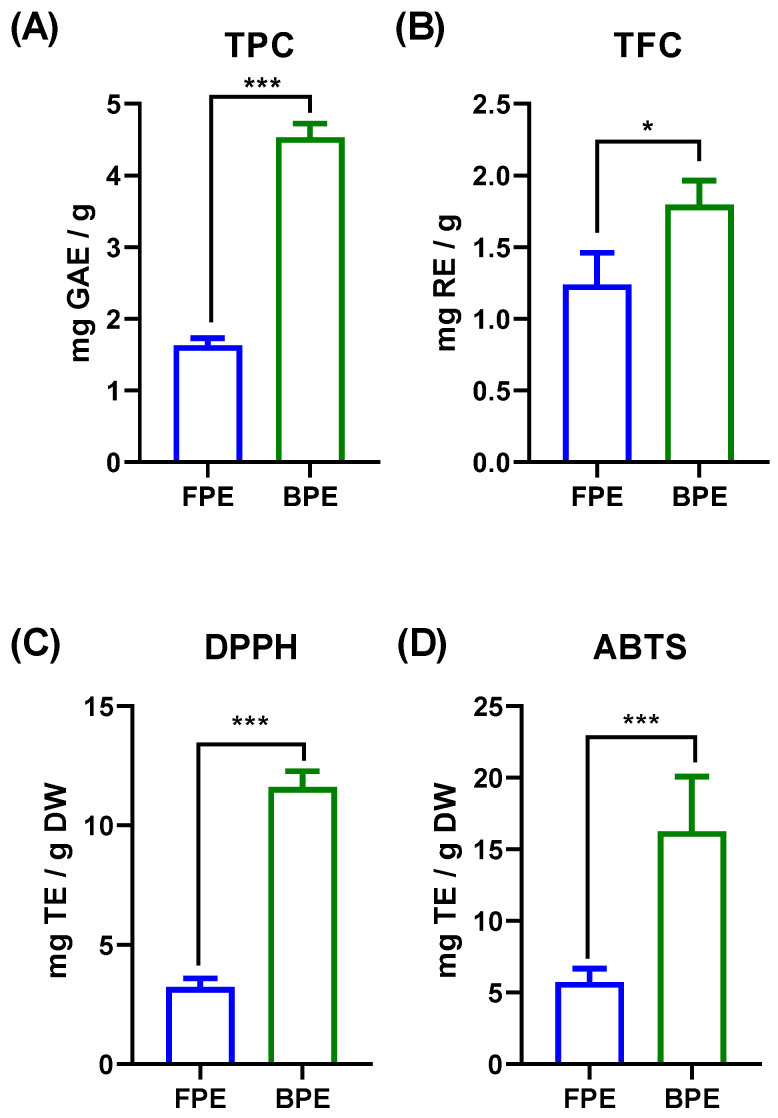
Total polyphenols content (**A**), total flavonoids content (**B**), antioxidant activities determined by DPPH (**C**) and ABTS assays (**D**) of FPE and BPE. Results are representative of three independent experiments and are expressed as mean ± SD. (*)(***) *p* < 0.05, 0.001, two-tailed Student’s *t*-test.

The TPC and TFC of FPE and BPE are shown in Figure 1. The results of the Folin–Ciocalteu assay showed that the TPC of FPE and BPE were 1.710 ± 0.031 mg GAE/g and 4.717 ± 0.085 mg GAE/g, respectively. The TFC of FPE and BPE were 1.272 ± 0.230 mg RE/g and 1.721 ± 0.205 mg RE/g, respectively. Previous studies reported that the TPC of darker colored quinoa seeds was higher than that of white quinoa seeds [10,15]. Tang et al. compared the TPC and TFC in quinoa seeds with different colors and their results showed that the TPC of red quinoa seeds (about 4.3 mg GAE/g) was higher than that of white quinoa seeds (about 2.1 mg GAE/g) [15]. Furthermore, our results also showed that the TPC and TFC of BPE were significantly higher than those in FPE (*p* < 0.05), which was in consistency with some previous findings that the content of bound polyphenols was largely distributed in cereal grains, such as rice (~62%), wheat (~75%), corn (~85%), etc. [16].

The chemical antioxidant activities of FPE and BPE were evaluated by the DPPH and ABTS assays. Both the DPPH and ABTS values of BPE (11.601 ± 0.669 mg TE/g DW, 16.251 ± 3.824 mg TE/g DW) were significantly higher than those of FPE (3.231 ± 0.361 mg TE/g DW, 5.732 ± 0.940 mg TE/g DW), which were in positive correlation with the TPC and TFC values. DPPH as a free radical has been widely used to evaluate the reducing molecules. ABTS is also frequently used in the food industry to determine the antioxidant activities of foods [17]. In comparison with cereal-based foods, both FPE and BPE exhibited stronger antioxidant activities [18]. Previous studies have shown that polyphenols are efficient hydrogen donors and electron transfer agents, which is in line with the principle of the DPPH and ABTS assays [19].

### 3.2. Components of FPE and BPE

**Figure 2:**
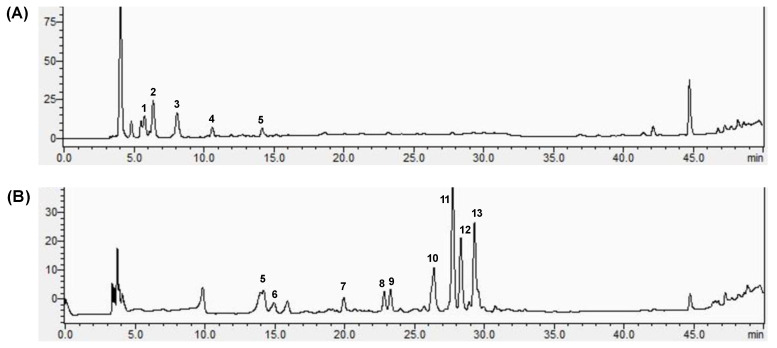
HPLC chromatogram of FPE (**A**) and BPE (**B**) from red quinoa. Peak 1: 2-hydroxybenzoic acid (tentatively identified); peak 2: vanillin; peak 3: caffeic acid; peak 4: gallic acid; peak 5: 3,4-dihydroxy-benzoic acid; peak 6: vanillic acid; peak 7: catechin; peak 8: chlorogenic acid; peak 9: epicatechin; peak 10: p-Coumaric acid; peak 11: ferulic acid; peak 12: ferulic acid 4-glucoside (tentatively identified); peak 13: quercetin.

The components of FPE and BPE were further analyzed via HPLC-MS (Figure 2). Table 1 shows the identification and quantification of the major peaks presented in Figure 2, with their retention times, mass spectral data and contents. As presented, a total of 13 polyphenols were identified, while the components of FPE and BPE varied a lot. Based on the mass spectrum and the comparison with commercial standards, the major phenolic components of FPE were 2-hydroxybenzoic acid, vanillin, caffeic acid, gallic acid and 3,4-dihydroxy-benzoic acid. These components are commonly found in fruits, vegetables and cereal grains, as previously reported [20]. Alkaline and acid treatment released a considerable amount of bound polyphenols from red quinoa. Five types of phenolic acid (3,4-dihydroxy-benzoic acid, vanillic acid, chlorogenic acid, p-coumaric acid and ferulic acid), two types of flavan-3-ols (catechin and epicatechin), one type of flavonoid (quercetin) and one type of phenolic glycoside (ferulic acid 4-glucoside) were identified in BPE. Among them, ferulic acid, ferulic acid 4-glucoside and quercetin contributed to a large part of BPE, which was also in consistency with some previous studies. They revealed that ferulic acid and its derivatives were the most common components of bound polyphenols from some major cereal grains, such as rice, wheat, barley, sorghum, etc. [6].

### 3.3. Inhibitory Effects and Mechanism of FPE and BPE against α-Glucosidase

**Figure 3:**
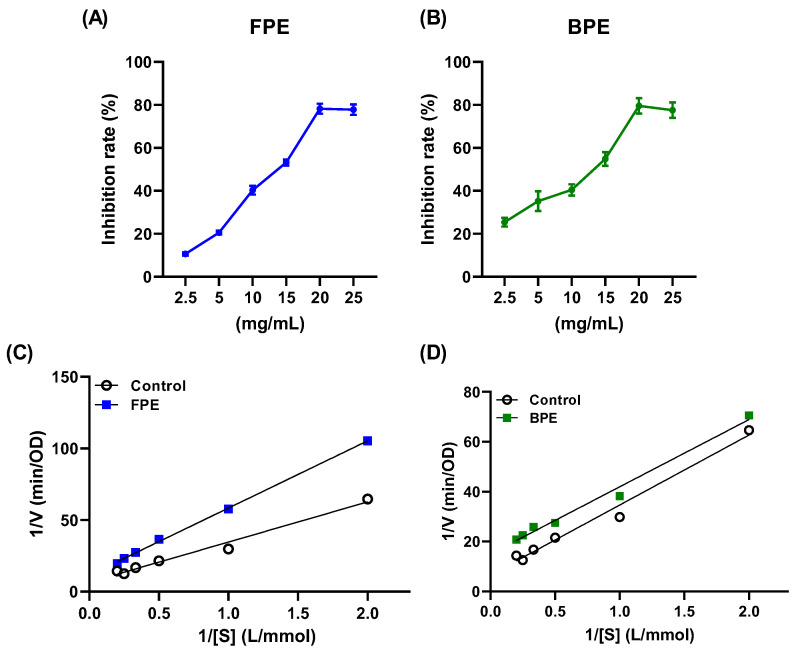
Inhibitory effects of FPE and BPE against α-glucosidase. Inhibition curve of FPE (**A**) and BPE (**B**) on α-glucosidase. Lineweaver–Burk plots of α-glucosidase inhibited by FPE (**C**) and BPE (**D**).

α-glucosidase, located in the brush border of the small intestine, hydrolyzes terminal non-reducing (1→4)-linked α-glucose residues to release a single α-glucose, which leads to an increase in blood glucose levels. The inhibitory effects of FPE and BPE were compared (Figure 3A,B). Both FPE and BPE inhibited the activity of α-glucosidase in a dose-dependent manner. The IC_50_ values of FPE and BPE were 13.013 ± 0.196 mg/mL and 10.295 ± 0.223 mg/mL, respectively. The IC_50_ value of BPE was lower than of FPE, indicating that the inhibitory effect of BPE on α-glucosidase was stronger than that of FPE. Both FPE and BPE exhibited higher α-glucosidase inhibitory activity than polyphenols from some other grains, such as corn and barley [21].

Reversible inhibitors can be classified into competitive, non-competitive, uncompetitive (or anti-competitive) and mixed types based on their interactions with the enzyme, which directly affect the values of Km and Vmax. Competitive inhibitors directly compete with the substrate to bind with the active site of the enzyme, while non-competitive inhibitors do not compete with the substrate and bind to the allosteric site of the enzyme instead. Uncompetitive inhibitors bind to the enzyme–substrate complex away from the active site of the enzyme, while mixed inhibitors bind to either the enzyme away from its active site or the enzyme–substrate complex [22]. The inhibition mode of α-glucosidase was determined from the Lineweaver–Burk plot, and the enzyme kinetic parameters following α-glucosidase inhibition with FPE and BPE are shown in Table 2. Figure 3C shows that the x-intercept was almost unchanged with a reduced Vmax value after adding FPE, which indicated that FPE inhibited α-glucosidase in a non-competitive mode. On the other hand, Figure 3D shows that after adding BPE, both the Km and the Vmax value decreased with two equations almost parallel to each other, indicating that BPE inhibited α-glucosidase in an uncompetitive mode. The components of FPE varied a lot from those of BPE, and this might have contributed to their different inhibition modes.

### 3.4. Effects of FPE and BPE on the Digestion of Rice Starch In Vitro

**Figure 4:**
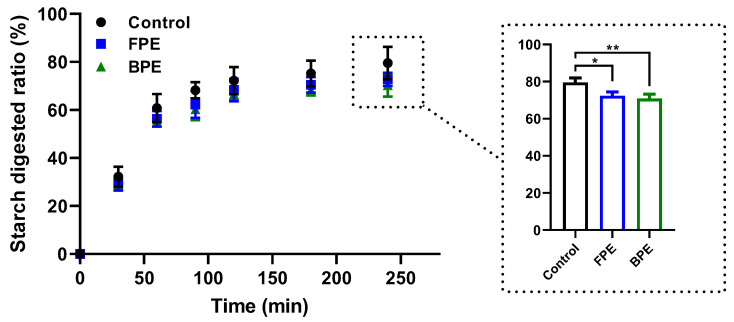
Effects of FPE and BPE on the digestion of rice starch in vitro. Results are expressed as mean ± SD (*n* = 3). (*)(**) *p* < 0.05, 0.01, compared with the control. Multiple comparisons were performed by one-way analysis of variance (ANOVA) followed by Tukey’s test.

The effects of FPE and BPE on the digestion of rice starch in vitro are shown in Figure 4. The digestion curves showed an exponential growth shape with a rapid digestion stage (0–60 min), followed by a much slower digestion phase afterwards until reaching the digestion plateau. At 240 min, the starch-digestion ratio of the control group reached 79.51%. The addition of FPE and BPE inhibited the starch digestion process to some extent. The starch digestion ratios were 72.74% and 70.28% at 240 min after the addition of FPE and BPE at 10 mg/mL, respectively, showing significant differences compared with the control (*p* < 0.05). In consistency with previous studies, BPE with the highest level of TPC, TFC and various components of polyphenols prevailed over FPE in terms of inhibiting starch digestion.

### 3.5. Effects of FPE and BPE on Postprandial Blood Glucose Levels In Vivo

**Figure 5:**
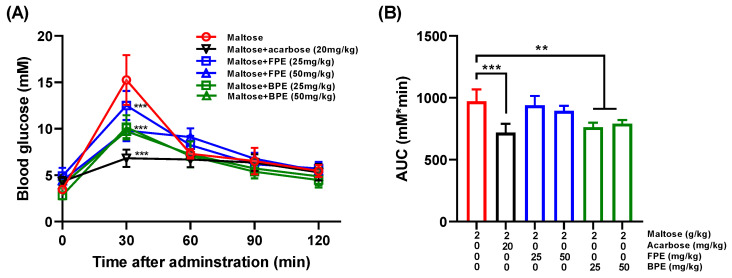
Effects of FPE and BPE on postprandial blood glucose levels in vivo. (**A**) Blood glucose levels after oral administration of maltose and acarbose, FPE and BPE (*n* = 5). (**B**) Area Under the Curve (AUC) after oral administration of maltose and acarbose, FPE and BPE for 2 h. Results are expressed as mean ± SD (*n* = 5). (**)(***) *p* < 0.01, 0.001, compared with the maltose group. Multiple comparisons were performed by one-way analysis of variance (ANOVA) followed by Tukey’s test.

To further explore the inhibitory effects of FPE and BPE on α-glucosidase in vivo, an oral maltose tolerance test was performed in a normal ICR mice model. Concentrations of 2g/kg of maltose were administered to the mice. In addition, 20 mg/kg of acarbose was used as a positive control. As presented in Figure 5A, the blood glucose level of all mice increased quickly in 30 min after maltose administration (about 15.25 mM), and gradually decreased afterwards. The rapid turbulence of blood glucose was improved by the administration of acarbose, FPE and BPE. At 30 min, the blood glucose levels of the maltose+acarbose group (20 mg/kg), the maltose+FPE group (50 mg/kg) and the maltose+BPE group (50 mg/kg) were 6.83 mM, 9.80 mM and 9.74 mM, respectively. The Area Under the Curve (AUC) was derived from the oral maltose tolerance test (Figure 5B). Administration of acarbose and BPE significantly reduced the AUC of mice (*p* < 0.05). Compared with the AUC of the maltose group, a 25.8% of reduction was observed in the maltose+acarbose group, and a 21.4% of reduction was observed in the maltose+BPE group (50 mg/kg). The suppressive effect of BPE at 50 mg/kg on postprandial hyperglycemia was comparable to that of acarbose at 20 mg/kg, indicating that BPE can be considered as a natural α-glucosidase inhibitor.

## 4. Discussion

T2DM has been a rapidly growing health problem in recent years. An estimated 850 billion USD was spent globally on the treatment and health interventions for diabetes in 2017 [23]. This number is expected to rise to 958 billion USD by 2045 [24]. Many clinical drugs have been invented to treat or manage T2DM by (a) stimulating the secretion of insulin and (b) interrupting or delaying the digestion of dietary starch to reduce the rate of blood sugar absorption from the small intestine [25]. Aiming at (b), acarbose and miglitol, acting as α-glucosidase inhibitors, were approved by the FDA to manage postprandial glucose rises. α-glucosidase is secreted in the small intestine and is found in the luminal surface of enterocytes. α-glucosidase is a key enzyme catalyzing the hydrolytic cleavage of disaccharides (maltose and sucrose) into monosaccharides (glucose and fructose). Thus, inhibiting the activity of α-glucosidase reduces the increasing of blood sugar and alleviates postprandial hyperglycemia [25]. However, side effects brought about by these drugs and high expenditure drive people to seek out effective plant-derived compounds serving as α-glucosidase inhibitors. 

Diets rich in polyphenols have been extensively studied in the past decade in terms of their effects on T2DM. A number of meta-analysis studies revealed that consuming polyphenol-enriched diets played a vital role in the prevention of T2DM, and this was found to be associated with lower risk of T2DM [26,27]. Some randomized controlled trails also revealed that the consumption of plant-derived polyphenols decreased fasting and post-prandial glucose and improved glucose metabolism in participants [28]. Polyphenols helped to manage T2DM in different ways, such as inhibiting digestive enzymes, regulating glucose metabolic pathways and modulating gut microbiota [29]. Similar to acarbose, some plant-derived polyphenols showed strong inhibitory effects on α-glucosidase and thus reduced the rate of digestion of complex starches, oligo-, tri- and disaccharides into absorbable glucose [25]. In recent years, quinoa has attracted growing attention due to its rich nutrients and many health benefits [30]. Compared with fruits and vegetables, cereal grains contain a much higher content of bound polyphenols (about 60%), which have always been neglected in terms of their activities and health benefits [6]. Our results exhibited that the content of BPE in red quinoa was about 2.7 times as much as that of FPE (Figure 1). Compared with FPE, BPE not only showed higher chemical antioxidant activities, as determined by the DPPH and ABTS assays, but also had stronger inhibitory effects on α-glucosidase (IC_50_ of BPE < IC_50_ of FPE).

Compared with free polyphenols, studies on the enzyme-inhibitory activities of bound polyphenols were limited. Zheng et al. used acid/alkaline hydrolysis to extract bound polyphenols from mung bean skin and showed that it was mainly composed of caffeic acid and protocatechuic acid and could inhibit the activity of α-glucosidase [31]. Yan et al. released non-extractable polyphenols of green tea by acid hydrolysis, and found that non-extractable polyphenols exhibited α-glucosidase inhibitory effects in a noncompetitive manner, although such effects were weaker than those of extractable polyphenols of green tea [14]. Meanwhile, Dong et al. found that in vitro fermentation released bound polyphenols from carrot dietary fiber, which showed stronger antioxidant activity than before fermentation and exhibited α-glucosidase inhibitory activity that was equivalent to that of acarbose [32]. Our results were in line with some previous findings that bound polyphenols showed stronger antioxidant and α-glucosidase inhibitory activities than free polyphenols. Studying the activities of bound polyphenols still requires further efforts. Particularly, the comparison between free polyphenols and bound polyphenols in terms of their components and activities needs further exploration. 

The composition of polyphenols might contribute to the differences between BPE and FPE regarding their antioxidant and enzyme-inhibitory effects. HPLC-MS analysis showed that hydroxybenzoic acids were the major components of FPE, while ferulic acid, ferulic acid 4-glucoside and quercetin were the major components of BPE (Figure 2, Table 1). These components exhibited strong antioxidant activities and inhibitory effects on enzymes as single compounds in previous reports [25]. Ferulic acid, as a major component of BPE, in particular, was reported to exert equivalent activity to acarbose [32]. The inhibitory mechanisms of polyphenols in α-glucosidase might be due to the following reasons: (a) hydrogen bonds and hydrophobic forces are formed between polyphenols and the active sites of the enzyme [33]; (b) ferulic acid was reported to increase the α-helix and decrease the β-sheet of α-glucosidase and, thus, hamper the active site formation or prevent substrate binding to alter the activity of α-glucosidase; (c) molecular docking analysis demonstrated that ferulic acid was mainly surrounded by the amino acid residues Asp215, Glu277, Gln279, Arg 315, Glu411, Tyr 158, Phe 159 and Phe178 of α-glucosidase, and thus formed three hydrogen bonds (Asp215, Glu277, Tyr158) and three π–π T-shaped (Phe158, Phe178, and Tyr 158) interactions. This further perturbed the protein structure of α-glucosidase [34]. Furthermore, different components of FPE and BPE also induced their different inhibition modes on α-glucosidase. The Lineweaver–Burk model indicated that FPE might bind to the allosteric site of α-glucosidase (non-competitive mode), while BPE might bind to the α-glucosidase-pNPG complex away from the active site of α-glucosidase instead (uncompetitive mode). Interactions between one polyphenol and the enzyme or one polyphenol and another comprehensively affect the binding sites to the enzyme or the enzyme–substrate complex. 

To further evaluate the outcome of the inhibitory effects of FPE and BPE on α-glucosidase, an in vitro starch digestion assay (Figure 4) and an in vivo postprandial blood glucose test (Figure 5) were carried out. The in vitro starch digestion assay showed that both FPE and BPE inhibited the process of starch digestion, and BPE delayed starch digestion to a larger extent compared with FPE. Furthermore, the results from the in vivo postprandial blood glucose test were in line with the in vitro starch digestion assay, showing that BPE significantly reduced the AUC for postprandial blood glucose compared with the maltose group. The effect of BPE at a higher dose (50 mg/kg) was comparable to that of acarbose (20 mg/kg). By inhibiting the activity of α-glucosidase in an uncompetitive mode, BPE effectively inhibited starch digestion in vitro and reduced the postprandial glucose increase in vivo.

## 5. Conclusions and Future Perspectives

In conclusion, the components, antioxidant activities, α-glucosidase inhibitory activity and mechanism, inhibition in starch digestion and in vivo postprandial glucose of FPE and BPE were investigated and compared in the present study. Alkaline and acid treatment released a large amount of bound polyphenols from red quinoa, and BPE showed higher levels of TPC and TFC than those of FPE. The HPLC-MS analysis revealed that the components between FPE and BPE varied greatly. Mainly composed of ferulic acid and its derivates, BPE showed stronger chemical antioxidant activities and α-glucosidase inhibitory activity. The Lineweaver–Burk model showed that FPE and BPE inhibited α-glucosidase activity in a non-competitive mode and an uncompetitive mode, respectively. By inhibiting α-glucosidase, BPE delayed starch digestion and suppressed postprandial hyperglycemia effectively. Bound polyphenols have been overlooked regarding their activities and application for a long time. Cereals grains consumed as staple foods all over the world contain rich bound polyphenols awaiting further investigation. Given that T2DM is prevailing around the world, exploiting functional components from cereal grains (staple foods) is necessary to manage T2DM. Our results demonstrate that BPE as a functional food has the potential to control postprandial glucose and, thus, is worth further investigation.

## Figures and Tables

**Table 1:** Components of FPE and BPE from red quinoa.

Peak	Retention Time (min)	Formula	[M-H]^−^ (*m*/*z*)	Identification	Content (mg/kg)	
					FPE	BPE
1	5.84	C_7_H_6_O_3_	137.03	2-hydroxybenzoic acid *	4.927 ± 1.023	ND
2	6.22	C_8_H_8_O_3_	151.04	Vanillin	3.170 ± 0.882	ND
3	8.04	C_9_H_8_O_4_	179.04	Caffeic acid	2.388 ± 0.673	ND
4	10.84	C_7_H_6_O_5_	169.01	Gallic acid	2.614 ± 0.541	ND
5	14.52	C_7_H_6_O_4_	153.02	3,4-dihydroxy-benzoic acid	2.774 ± 0.696	4.866 ± 0.334
6	15.98	C_8_H_8_O_4_	167.04	Vanillic acid	ND	2.615 ± 0.272
7	19.98	C_15_H_14_O_6_	289.07	Catechin	ND	2.788 ± 0.468
8	22.76	C_16_H_18_O_9_	353.09	Chlorogenic acid	ND	3.387 ± 0.668
9	23.22	C_15_H_14_O_6_	289.07	Epicatechin	ND	3.723 ± 0.334
10	26.46	C_9_H_8_O_3_	163.04	p-Coumaric acid	ND	9.753 ± 0.268
11	27.66	C_10_H_10_O_4_	193.05	Ferulic acid	ND	20.938 ± 3.866
12	28.44	C_16_H_20_O_9_	355.02	Ferulic acid 4-glucoside *	ND	11.794 ± 1.225
13	29.32	C_15_H_10_O_7_	301.04	Quercetin	ND	18.048 ± 1.236

* Peak 1 was tentatively identified as 2-hydroxybenzoic acid and was quantified as gallic acid equivalent. Peak 12 was tentatively identified as ferulic acid 4-glucoside and was quantified as ferulic acid equivalent. Values are expressed as mg/kg quinoa (mean ± SD) (*n* = 3).

**Table 2:** Enzyme kinetic parameters following α-glucosidase inhibition with FPE and BPE.

Samples	Double Reciprocal Equation	*K_m_* (mmol/L)	*V_max_* (OD/min)	R^2^
No inhibitor	y = 28.046x + 6.602	4.248	0.151	0.9821
FPE (20 mg/mL)	y = 46.893x + 11.507	4.075	0.087	0.9991
BPE (20 mg/mL)	y = 26.946x + 15.011	1.795	0.067	0.9874

## Data Availability

Exclude this statement.
